# How COVID-19 related psycho-social stressors affect longevity

**DOI:** 10.1192/j.eurpsy.2021.81

**Published:** 2021-08-13

**Authors:** D. Moser

**Affiliations:** 1 Child And Adolescent Psychiatry, Lausanne University Hospital CHUV, Lausanne, Switzerland; 2 Institute Of Psychology, University of Bern, Bern, Switzerland

**Keywords:** Psychosocial Stress, Projection Studies, Covid, Longevity

## Abstract

**Introduction:**

Before the COVID-19 pandemic, the literature on psychosocial stressors and psycho-social protective factors already clearly indicated that the two were linked in a multitude of ways to longevity. These ways include 1) directly through increased risk in suicides with respect to psycho-social stress or lack of connectivity 2) increased risk for psychopathologies such as depression, post-traumatic stress disorder and others, which in turn can decrease longevity in indirectly, and 3) a worse/healthier lifestyle that may be associated through decreased/improved social connectivity. With the advent of the COVID-19 pandemic, the ways in which these psychosocial factors could be impacted by policy came into focus. Attempting to quantify the potential future impact of such policies on longevity through psycho-social changes appeared necessary to allow better guidance of policy making. **Objective:** This presentation aims to leverage the experience gained from making a projection of the impact of pandemic mitigation strategies on longevity in the early advent of the COVID-19 pandemic.

**Results:**

The authors model indicated the high need for measures that are protective of the general populations’ psychosocial health in the face of a pandemic and associated mitigation strategies. **Discussion:** This presentation will discuss issues concerning quantifications of the impact of COVID-19 related policy on psychosocial health. The assumptions necessary to arrive at projective models may be at odds with parts of the current culture in the field. The presentation will discuss potential strategies in order for the scientific community to be better prepared for similar events in the future.

**Disclosure:**

No significant relationships.

